# A challenging diagnosis of malignant mesothelioma with osteosarcomatous differentiation metastasizing to bone

**DOI:** 10.1002/rcr2.664

**Published:** 2020-09-19

**Authors:** Michael Brown, Hubertus Jersmann, Thomas Crowhurst, Chris Van Vliet, Gareth Crouch, Arash Badiei

**Affiliations:** ^1^ Department of Thoracic Medicine Royal Adelaide Hospital Adelaide Australia; ^2^ Faculty of Health and Medical Sciences, Adelaide Medical School University of Adelaide Adelaide Australia; ^3^ Department of Anatomical Pathology PathWest Laboratory Medicine, QEII Medical Centre Nedlands Western Australia Australia; ^4^ Department of Cardiothoracic Surgery Royal Adelaide Hospital Adelaide Australia

**Keywords:** Mesothelioma, metastasis, pleura, pleural effusion, thoracoscopy

## Abstract

Malignant pleural mesothelioma (MPM) is an insidious primary neoplasm of the pleura that can be challenging to diagnose and is commonly considered to be only locally invasive. We present the case of a 74‐year‐old male who presented with clinical features of MPM but from whom pleural fluid and biopsies initially suggested benign pathology. He later developed diffuse bony metastases and re‐examination of pleural biopsies using modern immunohistochemistry and molecular testing revealed a diagnosis of sarcomatoid and desmoplastic MPM with heterologous osteosarcomatous differentiation. This case not only demonstrates the rare potential of skeletal metastasis of MPM, but also highlights the importance of recognizing the utility of modern diagnostic tests and their potential to prevent the need for unnecessary invasive procedures. To our knowledge this is the first description of this rare histological sub‐type presenting with skeletal metastases.

## Graphical abstract

We present a case of bony metastasis from a rare histological subgroup of malignant pleural mesothelioma. The diagnosis was challenging; however, modern immunohistochemistry and molecular testing provided a diagnostic opportunity and potentially saved further unnecessary investigation.

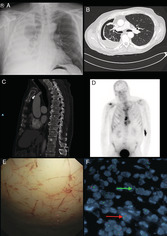

## Introduction

Malignant mesotheliomas are rare neoplasms arising from mesothelial surfaces (pleura, peritonea, tunica vaginalis, or pericardium). Malignant pleural mesothelioma (MPM) is the most common primary tumour of the pleura. Although traditionally associated with local spread and invasion, distant metastases to bone can rarely occur. Diagnostically, these tumours are challenging, either due to difficulties obtaining adequate malignant cellular material or similarity to adenocarcinoma. Some MPM sub‐types, such as epitheloid MPM, can be diagnosed from pleural fluid cytology. Rarer sub‐types like desmoplastic MPM are more challenging. We present a patient with clinical features of MPM whose pleural fluid cytology and pleural biopsies favoured a benign process despite multiple invasive procedures. Months later, the patient developed bony metastases. Biopsy of a metastasis and re‐examination of the original pleural biopsies with modern immunohistochemistry and molecular testing diagnosed combined sarcomatoid and desmoplastic MPM with heterologous osteosarcomatous differentiation. To our knowledge, this is the first description of this histological sub‐type presenting with skeletal metastases.

## Case Report

A 74‐year‐old reformed smoker (five pack‐years) presented with progressive dyspnoea over twelve months. He had immigrated to Australia from the United Kingdom in 1968. His occupations included working as an airline apprentice and industrial engraver. He had 18‐months of asbestos exposure as a maintenance fitter in the 1960s, cutting asbestos sheets. There was no personal history of lung disease or malignancy. His brother was a carpenter and died of mesothelioma. His father was a stone worker who died from gastric cancer.

**Figure 1:**
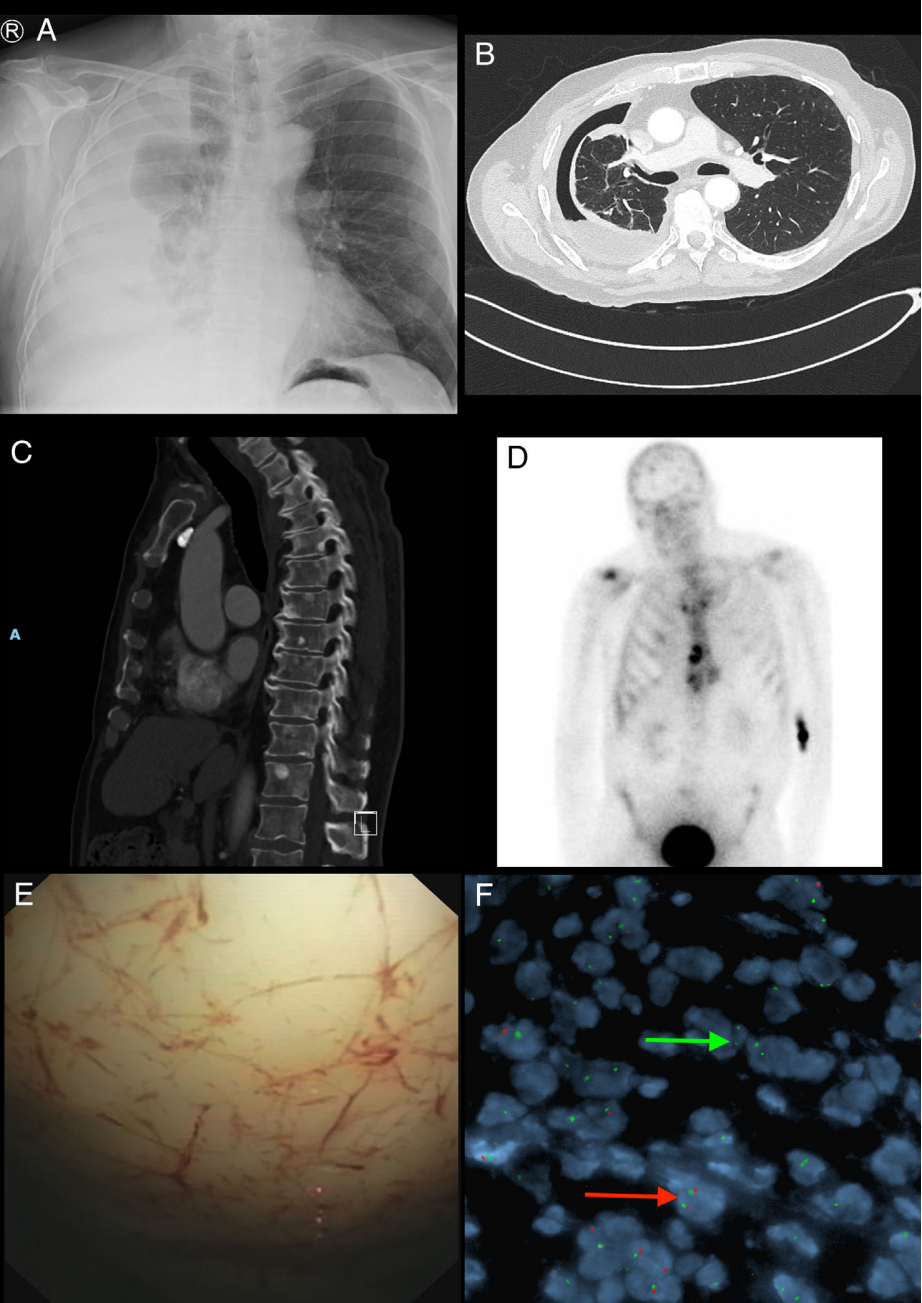
(A) Chest X‐ray showing a large right‐sided pleural effusion with lung collapse; (B) computed tomography (CT) chest demonstrating non‐expansible/trapped lung and pleural thickening; (C) CT chest showing vertebral metastases; (D) bone scan showing widespread osteoblastic metastasis to skull, vertebrae, ribs, sternum, and iliac crests; (E) pleuroscopy view of diffusely thickened pleura; (F) fluorescence in‐situ hybridization: green arrow shows abnormal nuclei with homozygous deletion of *CDKN2A* at 9p21 (absent red signals in the presence of two green chromosome 9 centromere signals). Red arrow shows nuclei with intact *CDKN2A* (two red target signals and two green chromosome 9 centromere signals).

A right‐sided pleural effusion was identified (Fig. 1A). Thoracentesis revealed blood‐stained, exudative pleural fluid with negative cytology. Subsequent chest computed tomography (CT) showed non‐expansible lung due to thickening of the visceral pleura (Fig. 1B). A uniport pleuroscopy with parietal pleural biopsies was performed (Fig. 1E). Histology demonstrated mild cytological atypia favoured to represent fibrinous pleuritis.

One‐month later, persisting clinical concern and pleural effusion recurrence, prompted a right thoracotomy with parietal and visceral pleural biopsies and a lung wedge biopsy. The visceral rind was notably invading into the underlying lung. The initial pathology also favoured benign pleuritis and morphologic and immunohistochemical evaluation did not establish a malignant neoplasm. The lung wedge was deemed non‐contributory, although the biopsy consisted of parenchymal hyalinised nodules with spindle cell proliferations. Breast cancer susceptibility gene 1 (BRCA1)‐associated protein‐1 (BAP‐1) staining was performed and did not demonstrate BAP‐1 loss.

Six months later, the patient remained well, but had required two further chest drains and insertion of an indwelling tunnelled pleural catheter for recurrent effusions. Given ongoing clinical and radiological concern for MPM, the treating team sought a second opinion on the surgical sample with fluorescence in situ hybridization (FISH) analysis from a pathology centre with MPM diagnostic expertise.

This pathology assessment reported a hypocellular spindle cell proliferation embedded within a rind of dense fibrosclerotic tissue. The spindle cells were arranged in cords, short fascicles, and storiform patterns. One cellular foci was intimately associated with osteoid. A pleural‐based nodule with a hyalinized central zone with spindle cells and storiform pattern peripherally was identified. Immunohistochemistry showed that the spindle cells expressed Cytokeratin (CK) AE1 + 3 and D2‐40 with limited staining for CK5/6 and calretinin. BAP‐1 expression was preserved and there was no staining for Wilms' Tumour 1 (WT1). Interphase FISH studies showed homozygous loss of cyclin‐dependent kinase inhibitor 2A (*CDKN2A*) at 9p21 in three separate biopsy samples, including the pleura and wedge specimens (Fig. 1F). A diagnosis of sarcomatoid and desmoplastic mesothelioma with heterologous osteosarcomatous differentiation was made. The patient declined chemotherapy.

Nine months after his initial investigations, CT chest demonstrated widespread lytic lesions of the right ribs and sternum and innumerable sclerotic lesions within the spine (Fig. 1C). Whole body bone scan showed widespread osteoblastic metastases (Fig. 1D). CT‐guided biopsy of the sternum showed features consistent with metastatic MPM with sarcomatoid and desmoplastic patterns.

## Discussion

MPM has an incidence in Australia of 2.8 per 100,000 with a male–female ratio of 4:1 (1). The number of cases has been rising, correlating with high per capita asbestos exposure, which has an aetiological fraction above 80% (2). Asbestos use peaked in the 1970s and declined until its ban in 2003. The long latency between exposure and disease means the incidence of MPM has only recently plateaued in Australia (1).

MPM presents non‐specifically with chest pain, dyspnoea, and cough. Pleural effusion is common. Rarely, MPM presents with local invasion or distant metastases. The prognosis is poor due to its aggressive natural history, insidious presentation, diagnostic complexity, as well as the paucity of treatment options. Survival depends on the mesothelioma type, which is categorized by the World Health Organization (WHO) into three major subtypes. Epithelioid is the most common variant, followed by sarcomatoid and biphasic, which contains both sarcomatoid and epithelioid areas. Desmoplastic mesothelioma is a subtype of the sarcomatoid form (3). Rusch et al. analysed 3101 patients and reported median survival times at 19 months for epithelioid, 13 months for biphasic, and eight months for sarcomatoid tumours (4). Poor survival rates particularly for sarcomatous mesothelioma are documented in other studies with survival rates closer to six months and as low as 3.5 months (5). Cantin et al. analysed a desmoplastic subgroup and of their 26 patients the median survival was 5.0 months (6).

Diagnosing mesothelioma is challenging, often requiring multiple invasive pleural procedures. Recent advances in cytological and histological testing techniques have improved diagnostic pathways. Pleural effusion often provides the first opportunity for cytological analysis and this can be an adequate diagnostic test without needing further invasive tests, particularly for epithelioid and biphasic MPM (7). Sarcomatoid MPM is more difficult to diagnose because cells are not exfoliated into the pleural fluid and hence additional invasive tests are often required. The International Mesothelioma Interest Group (IMIG), endorsed by the International Academy of Cytology and the Papanicolaou Society of Cytopathology, have released diagnostic criteria for the diagnosis of epithelioid and biphasic MPM in pleural fluid (8). The sensitivity and positive predictive value for pleural fluid cytology in diagnosing MPM is reported as 86% and 99%, respectively (9). If pleural fluid cytology is negative but clinical suspicion persists, tissue biopsy should be pursued to identify characteristic histological features, which are outlined in the WHO classification (3, 10).

A diagnostic challenge is differentiating MPM from adenocarcinoma and mesothelial fibrinous pleuritis. Immunohistochemistry is particularly important and can be performed on cytological (including pleural fluid) or histological specimens. The immunohistochemical markers reflected in the IMIG and WHO guidelines for tumours of mesothelial origin are calretinin, CK5/6, WT1, D2‐40, and mesothelin. Epithelial markers include claudin 4, CEA, MOC31, TAG‐72, Ber‐EP4, and MUC4 (3, 10, 11).

Recently, BAP‐1 immunohistochemistry has been recognized as a useful ancillary test in diagnosing mesothelioma. BAP‐1 protein loss in MPM ranges from 56% to 68% (12) and is particularly common in epithelioid tumours rather than sarcomatoid subtypes (13). There are no current reports of BAP‐1 protein loss in benign mesothelial proliferations; therefore, it is a useful marker to differentiate benign versus malignant (14, 15).

FISH testing for *CDKN2A* is an additional test that should be performed if the diagnosis is uncertain; this was a critical diagnostic test in our case. Homozygous deletion of *CDKN2A* occurs in all forms of MPM with prevalence of 67–100% in sarcomatoid, 69–95% in biphasic and 48–70% in epithelioid subtypes (13). Importantly, *CDKN2A* FISH can reliably distinguish between sarcomatoid mesothelioma and fibrinous pleuritis (16).

In combination, BAP‐1 immunohistochemistry and *CDKN2A* FISH analysis have 100% specificity for malignant mesothelioma (17, 18). Hwang et al. reported a combined sensitivity of 85% for sarcomatoid and desmoplastic MPM (19). Hence, these tests should be pursued in challenging cases when clinical suspicion is high and the diagnosis remains uncertain after traditional analysis.

Metastatic MPM is a rare but well‐documented phenomenon often only recognized at autopsy. A post‐mortem study of 318 patients with MPM identified distant metastases in 55.4% of cases, including the liver (31.9%), bone (13.8%), spleen (10.8%), thyroid (6.9%), and brain (3.0%) (20). The rate of bone metastasis is documented similarly in other studies (21). Cantin et al. published a case series looking specifically at the distant spread of desmoplastic mesothelioma and of 27 autopsy cases, 11 had distant metastases to lymph nodes (33.3%), liver (27.8%), lung (16.6%), adrenal glands (16.6%), and kidneys (16.6%) (6). Other metastatic sites for MPM have been described, including cutaneous and breast metastases (22, 23), bowel (24), oral cavity (25), and the central nervous system (26).

Case reports of distant skeletal metastases are uncommon. We identified 18 in the English literature (Table 1). Skeletal metastases were more common in males (79%) and in those with biphasic sub‐types (six cases). This was followed by desmoplastic (five cases), epithelioid (three cases), mesenchymal and sarcomatoid mesothelioma (one case each) subtypes. The most common site was the vertebra (56% of cases). Long bone involvement was seen in 28% of cases.

**Table 1:** Identified case reports of metastatic malignant mesothelioma to bone in the English literature.

Author	Year	Age	Sex	Histology	Site of metastases
Ihara et al. (27)	2018	64	M	Epithelioid	Bone marrow, thoracic spine, lumbar spine, and sacral vertebra. Spinal cord compression at T11/12
Arslan et al. (28)	2016	59	M	Epithelioid	Retromolar trigone, C7 vertebra
Knipscheer et al. (29)	2013	64	M	Biphasic	Left humerus, bone marrow
	2013	64	M	Mesenchymal	Thoracic vertebrae, bone marrow
Lester and Xu (30)	2008	76	F	Biphasic	Right femoral neck
Terakado et al. (31)	2004	53	M	Sarcomatoid	Mandible, small intestine
Swayne et al. (32)	1992	70	F	Biphasic	Right acetabulum
Cheng and Berkman (33)	1990	71	M	Biphasic	Pericardium, thoracolumbar vertebrae and abdominal organs
Machin et al. (34)	1988	78	M	Desmoplastic	Pelvis and vertebrae
	1988	70	M	Desmoplastic	Both humeri, femurs, 8th rib bilaterally
Cedrés et al. (35)	2013	67	M	Epithelioid	Thoracic and lumbar vertebrae and nerve root involvement compressing L3
Laurini (36)	1974	56	M	Biphasic	Right head of humerus, C7 to L5 vertebrae, pericardium
Hirano et al. (37)	2003	77	M	Desmoplastic	Thoracic vertebrae, meninges and liver (found on autopsy)
Ishikawa et al. (38)	2003	82	F	Desmoplastic	9th and 10th thoracic vertebrae (found on autopsy)
Hayashi et al. (39)	2010	70	M	Biphasic	Stomach, “spine and breast bone”
Huang et al. (40)	2009	64	M	NA	T11 vertebra
Singh et al. (41)	2013	58	F	NA	Left femur
Moskowitz et al. (42)	2016	71	M	Desmoplastic	Iliac crests, calvarium and clivus

Heterologous osteosarcomatous differentiation is rare. Klebe et al. presented 27 cases of heterologous differentiation and reviewed another 23 published cases (43). Twelve of these cases were previously described by Yousem and Hochholzer (44). Itano et al. have also described the literature and a case of sarcomatoid pleural mesothelioma with osteosarcomatous differentiation (45). Including this case, 56 cases of heterologous pleural mesothelioma have currently been reported. In this group, metastatic disease was infrequently recorded. Goldstein reported lung, epicardium, and liver metastasis (46). Salgado et al. identified metastasis in the mediastinal nodes, vasculature, and pericardium (47). Shiba et al. referenced metastasis, though the location was not described (48). Itano et al. described pulmonary and intraperitoneal metastasis (45). To our knowledge, there have been no reports describing bony metastases in this histological sub‐type and hence we believe this is the first description of combined sarcomatoid and desmoplastic mesothelioma with osteosarcomatous differentiation metastasizing in this way.

MPM is a rare tumour of the pleura. Although commonly viewed as a locally invasive tumour, distant metastases are possible, but rare. We have described the first case of combined sarcomatoid and desmoplastic MPM with osteosarcomatous differentiation metastasizing to bone. This case also highlights the diagnostic challenges of mesothelioma, particularly in differentiating malignancy from fibrinous pleuritis. Employing modern immunohistochemistry assessing for BAP‐1 protein loss and FISH for homozygous deletion of *CDKN2A* can be invaluable and may avoid unnecessary invasive investigations.

### Disclosure Statement

Appropriate written informed consent was obtained for publication of this case report and accompanying images.
